# HEARING LOSS AND COGNITIVE DECLINE: POLICY RECOMMENDATIONS

**DOI:** 10.1093/geroni/igae098.3851

**Published:** 2024-12-31

**Authors:** Girish Hemrajani, Debra Dobbs

**Affiliations:** University of South Florida, Tampa, Florida, United States; University of South Florida, Tampa, Florida, United States

## Abstract

Hearing loss is the third most common health condition in older age after arthritis and heart disease. With a rapidly increasing older adult population, hearing loss cases are expected to grow from 38 million to 73 million by 2060. Hearing loss in midlife has been estimated to account for 9% of cases of dementia, a huge – but potentially reversible – disease burden given that dementia affects 47 million people worldwide.

**Methods:**

The systematic literature review was included from the Ageline database, containing articles published in English from January 2013 to June 2024. The search terms ‘hearing loss,’ ‘cognitive decline’ and ‘older adults’ produced 161 results and out of these 18 relevant articles were chosen for the review.

**Results:**

In eight articles, hearing interventions were found to reduce cognitive decline in cognitively healthy older adults with hearing loss and slow down the progression of cognitive decline. One article found the provision of hearing aids would have a 48% protective effect on cognitive changes compared to the control over three years. The other articles were equally divided as “not sure” and “need further investigations”. Implications: The review highlights the importance of addressing hearing loss as a preventative measure and suggests policy measures and prevention strategies to maximize cognitive well-being in older adults. Age related sensorineural hearing loss is widely treatable with hearing aids and targeted intervention for hearing loss may play a fundamental role in dementia prevention. Key words: hearing loss, cognitive decline, hearing aid.

